# Measles Virus Epitope Presentation by HLA: Novel Insights into Epitope Selection, Dominance, and Microvariation

**DOI:** 10.3389/fimmu.2015.00546

**Published:** 2015-11-02

**Authors:** Ingrid M. Schellens, Hugo D. Meiring, Ilka Hoof, Sanne N. Spijkers, Martien C. M. Poelen, Jacqueline A. M. van Gaans-van den Brink, Ana I. Costa, Harry Vennema, Can Keşmir, Debbie van Baarle, Cécile A. C. M. van Els

**Affiliations:** ^1^Centre for Infectious Disease Control, National Institute for Public Health and the Environment, Bilthoven, Netherlands; ^2^Laboratory of Translational Immunology, Department of Immunology, University Medical Center Utrecht, Utrecht, Netherlands; ^3^Department of Internal Medicine and Infectious Diseases, University Medical Center Utrecht, Utrecht, Netherlands; ^4^Institute for Translational Vaccinology, Bilthoven, Netherlands; ^5^Theoretical Biology and Bioinformatics, Utrecht University, Utrecht, Netherlands

**Keywords:** HLA class I, measles virus, CD8 T cell epitope, immunodominance, epitope mapping, HLA-A antigens, HLA-B antigens, HLA-C antigens

## Abstract

Immunity to infections with measles virus (MV) can involve vigorous human leukocyte antigen (HLA) class I-restricted CD8^+^ cytotoxic T cell (CTL) responses. MV, albeit regarded monotypic, is known to undergo molecular evolution across its RNA genome. To address which regions of the MV proteome are eligible for recognition by CD8^+^ CTLs and how different HLA class I loci contribute to the epitope display, we interrogated the naturally processed and presented MV peptidome extracted from cell lines expressing in total a broad panel of 16 different common HLA-A, -B, and -C molecules. The repertoire and abundance of MV peptides were *bona fide* identified by nanoHPLC–MS/MS. ­Eighty-nine MV peptides were discovered and assignment to an HLA-A, -B, or -C allele, based on HLA-peptide affinity prediction, was in most cases successful. Length variation and presentation by multiple HLA class I molecules was common in the MV peptidome. More than twice as many unique MV epitopes were found to be restricted by HLA-B than by HLA-A, while MV peptides with supra-abundant expression rates (>5,000 cc) were rather associated with HLA-A and HLA-C. In total, 59 regions across the whole MV proteome were identified as targeted by HLA class I. Sequence coverage by epitopes was highest for internal proteins transcribed from the *MV-P/V/C* and *-M* genes and for hemagglutinin. At the genome level, the majority of the HLA class I-selected MV epitopes represented codons having a higher non-synonymous mutation rate than silent mutation rate, as established by comparison of a set of 58 unique full length MV genomes. Interestingly, more molecular variation was seen for the epitopes expressed at rates ≥1,000 cc. These data for the first time indicate that HLA class I broadly samples the MV proteome and that CTL pressure may contribute to the genomic evolution of MV.

## Introduction

Measles is a highly contagious disease, caused by the measles virus (MV), an enveloped single-stranded Morbillivirus. MV has no animal reservoir and can only be maintained in human populations by a continuous chain of acute infections. A single attack of measles as well as receiving two doses of an attenuated MV vaccine confer life-long immune protection (1). This is remarkably different from many other viruses that can cause (re-)infections within several years by readily adapting to the host immune response. MV is regarded serologically monotypic although genomic variation is observed across the RNA genome. Based on comparison of genes encoding envelope proteins that are suspected to encounter immune pressure from neutralizing antibodies, MV molecular evolution is considered modest relative to other viruses (2, 3). This may suggest that specific immune responses to MV are relatively modest, yet, on the contrary, MV evokes considerable immune responsiveness, underlying the host’s long-lived protection (4, 5). At the same time, and this is often regarded as the “measles paradox,” the acute phase of measles is also associated with strong immune suppression for up to months, which causes significant secondary morbidity and mortality (6, 7). Viral mechanisms have been proposed to account for this, including MV infection of dendritic cells (6, 8, 9), and more recently, infection of CD150 positive memory T cells and follicular B cells (10). Previously, we have pointed out a possible molecular mechanism connecting vigorous MV-specific immunity to the observed immune suppression (4). A single viral peptide was found to dominate the peptide repertoire presented at the surface of a MV-infected human B lymphoblastoid cell line (BLCL) in the context of the human leukocyte antigen (HLA) class I molecule HLA-A2 (11). This supra-abundant peptide, spanning amino acids (aa) 84–92 from the non-structural C protein (MV-C_84–92_), induced vigorous CD8^+^ T cell expansions in acute measles patients to estimated peak fractions of 7.5–15% of specific CD8^+^ T cells (4). We postulated that such an epitope could account for CD8^+^ T cell-mediated mass destruction of infected immune cells in hosts with this particular HLA type. Notably, the apparent genetic variation in this epitope, abrogating its binding to the HLA-A2 molecule (11), indirectly suggested that effective anti-viral immunity is at play that may induce immune escape. Yet to account for the general immune suppression observed after measles, this phenomenon cannot be limited to a single epitope in HLA-A2 but should rather involve other HLA class I molecules as well. In principle, every HLA class I molecule on nucleated cells binds small peptides, generated by proteolysis from cytosolic proteins, and presents it as an HLA class I-peptide complex (HLAp) at the cell surface to CD8^+^ T cells. Which peptides are picked up by HLA-A, -B, and -C molecules in the class I loading pathway depends on their unique peptide binding motifs. These motifs and the available “degradome” determine the expression of a particular HLAp, which can range from 1 to 10, 100 to 1,000, or even from 10 to 100,000 copies per cell (cc) (11, 12). Recently, using advanced mass spectrometry technology, we found that repertoires of *self* peptides presented by a set of HLA-A alleles were less diverse than those presented by their co-expressed HLA-B alleles, allowing potentially more dominance by HLA-A peptide cargo (13). Here, we tested the hypothesis that HLA-A alleles favor the presentation of supra-abundant MV peptide species. We analyzed MV-specific HLAp repertoires presented 48 h after MV infection in the context of in total 16 different HLA-A, -B, and -C molecules.

## Materials and Methods

### Cell Lines and MV Infection

Four human BLCL, 1053, 1077, 1090, and 1112 (kindly provided by Dr. T. Mutis, VUmc, The Netherlands) were grown in RPMI-1640 medium supplemented with penicillin, streptomycin, and 10% heat-inactivated fetal calf serum (FCS). Together the BLCL express five different HLA-A, six different HLA-B, and five different HLA-C alleles (Table 1), covering >70 and >50% of the HLA-A- and HLA-B allele usage in the Caucasian population, respectively. Approximately 2 × 10^8^ cells of each BLCL were infected at an m.o.i of 0.5 for 48 h in RPMI-1640 medium supplemented with antibiotics and 2% FCS using plaque-purified MV (Edmonston B strain), propagated on Vero cells. This procedure yields >90% infected cells, based on expression of MV-Hemagglutinin (MV-H). Approximately 2 × 10^8^ cells of each BLCL were left uninfected. Infected and uninfected (control) BLCL batches were harvested, washed three times in cold PBS, pelleted, snap-frozen, and stored at −70°C until further use. Pre- and post-infection cell viability was high. MV infection did not affect the expression of total HLA class I molecules, of HLA-A*02 molecules nor of HLA-B and -C molecules as was assessed by flowcytometric analysis of HLA-typed BLCL using the monoclonal antibodies W6/32, BB7.2 (if applicable) and B1.23.2, respectively.

**Table 1:** **Summary of HLA class I alleles and number of identified MV peptides and source proteins per BLCL**.

Cell line	HLA-A	HLA-B	HLA-C	MV peptides	MV proteins represented
BLCL1053	A*02:01	B*07:02	C*07:02	27^a^ (13, 7, 2, 5)^b^	7
	A*03:01	B*07:02	C*07:02		
BLCL1077	A*01:01	B*08:01	C*03:04	12 (0, 12, 0, 0)	5
	A*24:02	B*40:01	C*07:01		
BLCL1090	A*02:01	B*35:01	C*04:01	22 (10, 10, 0, 2)	7
	A*11:01	B*44:02	C*05:01		
BLCL1112	A*02:01	B*15:01	C*03:04	28 (7, 11, 1, 9)	7
	A*02:01	B*44:02	C*05:01		
Total				89^c^^,^^d^ (30, 40, 3, 16)	8
Total unique	5	6	5	70^e^ (18, 38, 3, 11)	

*^a^Number of unique HLApMV combinations identified per cell line*.

*^b^Between brackets the number of assignments to HLA-A, -B, -C, and NA, respectively*.

*^c^Total number of HLApMV combinations identified in this study*.

*^d^A cumulative list of all identified HLApMV combinations with detailed information on source sample, allele assignment, and expression rate is given in Table S2 in Supplementary Material*.

*^e^Unique HLApMV combinations are short-listed in Table 2*.

### Isolation of HLA Class I-Bound Peptides

HLA class I-peptide complex were isolated from uninfected and 48 h MV-infected BLCL using W6/32 as described previously (11). Briefly, cells were solubilized in lysis buffer containing CHAPS and protease inhibitors. After centrifugation at 10,000 × *g* for 1 h at 4°C, supernatants were precleared with CNBr-activated and Tris-blocked control sepharose beads and beads coupled to normal mouse serum, respectively, and cleared with beads coupled to W6/32. Immunoprecipitated HLAp were eluted from the beads with 10% acetic acid and peptides were collected by passage over a 10-kDa MW cut-off membrane filter. The eight filtrated eluates were concentrated by vacuum centrifugation.

### Nanoscale Liquid Chromatography-Mass Spectrometry and Epitope Identification and Semiquantification

Each eluate was reconstituted in 0.1% (v/v) TFA and fractionated into 26 fractions using strong cation exchange (SCX) chromatography on a 200-μm I.D. PolySULFOETHYL Aspartide column (packed in-house), running a linear KCl-gradient starting with water in 0.5% (v/v) acetic acid to 500 mM and 35% (v/v) acetonitrile in 0.5% (v/v) acetic acid. Fractions were dried by vacuum centrifugation and reconstituted in water containing 5% formic acid and 5% dimethylsulfoxide. Peptide analyses on fractions using equivalents of ~50 × 10^6^ cells were performed on a nanoscale LC-MS system, essentially as described by Meiring et al. (14), comprising a 50- and 25-μm I.D. Reprosil-Pur C18-AQ trapping and analytical column, respectively (packed in-house). High-resolution MS1 data were acquired on an LTQ-Orbitrap XL mass spectrometer (Thermo Scientific, San Jose, CA, USA) at a resolution of 60,000 FWHM and CID MS/MS fragmentation spectra were acquired on-the-fly in the LTQ mass analyzer on the doubly and triply charged ions only. Peptide identification (with a false discovery rate of 5%) was performed with BioWorks 3.3.1 SP1 (Thermo Scientific, San Jose, CA, USA) against the human-annotated and MV-annotated proteins extracted from the UniProtKB/Swiss-Prot database (Swiss-Prot database version 57.10 with taxonomy identifier “Homo Sapiens” and “Measles Virus”, respectively)^1^. No enzyme cleavage specificity was used as a filter during the database search. Moreover, deamidation of N, oxidation of M, and phosphorylation of S, T, and/or Y were considered as dynamic modifications during the peptide identification process. Candidate peptides that were discovered in this process as MV-derived peptides were validated manually based on their MSMS spectrum and by confirmation of absence from the corresponding control eluate by comparative ion mapping. For semiquantification of peptide expression levels, known amounts of two internal standard peptides, Angiotensin-III and Oxytocin (Sigma-Aldrich, St Louis, MO, USA) were added to each of the SCX fractions and their response factors in the MS analysis (total counts under the peak curve/mole) were determined. Expression levels for the MV-derived peptides were calculated and given as copies per cell (cc), based on their counts in an SCX fraction (area under the curve) and based on the assumption of equal response factors for MV-derived peptides and the internal standard peptides. Experiments were performed as unique experiments due to their laborious nature.

### Peptide Assignment to HLA Class I Alleles

W6/32-isolated HLAp contain all groups of HLA class I molecules (HLA-A, -B, and -C). HLA molecules expressed per BLCL had little overlap in binding motifs, minimizing the level of cross-presentation (i.e., the presentation of the same peptide by two or more distinct HLA alleles). For each of the eluted MV peptides, an HLA class I allele was predicted using the NetMHC-3.2 algorithm (15, 16) for the respective BLCL’s HLA-A and -B alleles, and NetMHCpan-2.4 (17, 18) for HLA-C alleles, giving a binding prediction based on affinity scores. Because affinity scores for different HLA alleles may not necessarily be comparable, each peptide was also ranked among a set of 100,000 random natural peptides. A peptide was assigned to an HLA isotype (among all HLA isotypes expressed by a given BLCL) for which the peptide showed the highest ranking, while having a predicted affinity score <5,000 nM IC50. In addition, the ranking had to be among the top 5% for HLA-A and -B alleles and the top 10% for HLA-C alleles. We repeated the peptide assignment procedure also based on predicted binding affinity. A peptide was assigned to an HLA-C molecule only if the peptide did not rank among the top 5% for any of the respective BLCL’s HLA-A or -B isotypes, because prediction tools for the HLA-C locus do not reach the same prediction performance as for HLA-A and -B alleles. Peptides that failed to exceed the prediction score or ranking cut-off for any of the HLA-A, -B, and -C alleles remained “non-assigned” (NA). The assignment approach was validated by consolidating *self* peptide repertoires assigned to HLA-B*40:01 and -B*4402 by Hillen et al. (19) in our *self* peptide dataset (13).

### MV Sequence Alignment and Variability Analysis

Nucleotide gene sequences for nucleoprotein (MV-N), phospoprotein (MV-P), V protein (MV-V), C protein (MV-C), matrix protein (MV-M), fusion protein (MV-F), hemagglutinin (MV-H), and polymerase (MV-L) from 58 unique full-length sequenced MV genomes were obtained from Genbank (Table S1 in Supplementary Material), aligned, and translated using BioEdit (version 7.2.4) software. SNAP v2.1.1 software^2^ was used for codon analysis and calculation of non-synonymous and synonymous substitution rates, *dN* and *dS*, respectively.

### Statistical Analysis

For data analysis and visualization of data GraphPad Prism (Version 6.05) was used. To test associations between the proportion of codons with *dN* > *dS* of total epitope codons with either HLA class I locus (HLA-A versus HLA-B) or with epitope abundance (<1,000 versus ≥1,000 cc), the Fisher’s exact contingency test was used (Table S4 in Supplementary Material). *P*–values <0.05 were considered statistically significant.

## Results

### High-Resolution MV Peptide Identification by Mass Spectrometry

Peptide repertoires were eluted from HLA class I molecules expressed on MV-infected BLCL1053, -1077, -1090, and -1112, fractionated, analyzed by high-resolution nano-LC-MS and mass sequencing, and searched for the presence human and MV sequences. As expected, each eluate contained thousands of distinct peptides, mostly *self* peptides (13). Each eluate also contained multiple peptides unique for MV, based on peptide fragmentation and comparative ion mapping in control samples. As an example, ion 537.295 Da (MH^2+^), present in the eluate of MV infected but not of uninfected BLCL1112 (Figure S1 in Supplementary Material, lower versus upper panel, respectively), was identified by mass sequencing as KIIDNTEQL. This peptide represented aa 204–212 from the MV-M (M.204.09) (Figure S1 in Supplementary Material). Collectively in the four post-infection eluates, 89 MV-specific peptide identifications were made (Table 1).

### Assignment of HLA Class I Alleles and Expression Rate of the Identified MV Peptidome

Based on HLAp affinity prediction (see Materials and Methods) KIIDNTEQL peptide described above was assigned as a ligand of HLA-A*02:01, one of the expressed HLA class I molecules of BLCL1112. In addition, the expression rate of M.204.09, i.e., the number of HLA-A*02:01 molecules on BLCL1112 cells loaded with KIIDNTEQL, was semi quantitated using internal standard peptides (see Materials and Methods) (4,666 cc). Similarly, all 89 MV peptides identified in this study were allocated to their best predicted HLA class I molecule and semi quantitated. Thirty eluted MV peptide sequences were allocated to HLA-A alleles, 40 to HLA-B alleles, 3 to an HLA-C allele, and 16 could not be conclusively assigned to any single HLA molecule expressed by our BLCL (NA) (Table 1, details are given in Table S2 in Supplementary Material). Due to a partial overlap in HLA class I isotypes between BLCLs in this study, the same HLA-class I-MV peptide (HLApMV) combination could be found in peptide repertoires from two or three BLCL, as was observed for eight and four MV peptides, respectively. In three cases, a MV peptide had a length variant, discovered in the same eluate and assigned to the same allele. Such length analogs can be considered as representing the same MV epitope. After these considerations, 70 unique HLApMV combinations were identified in this study, 54% (*n* = 38) being assigned to HLA-B alleles, 26% (*n* = 18) to HLA-A alleles, 4% (*n* = 3) to HLA-C, and 16% (*n* = 11) remaining NA, respectively (Table 1). The HLApMV short-list represents mostly novel human HLA class I presented MV epitopes, with the exception of only four epitopes reported as CD8^+^ T cell targets earlier (Table 2) (4, 11, 20–24).

**Table 2:** **Cumulative list of unique eluted HLApMV combinations**.

Unique HLApMV	MV epitope sequence	MV epitope code^a^	Best HLA class I allele	Reference
1	YPALGLHEF	N.281.09	HLA-B*07:02	This study
2	YPALGLHEF	N.281.09	HLA-B*35:01	This study
3	GPRQAQVSF(L)	N.411.09 (10)	HLA-B*07:02	This study
4	DALLRLQAM	N.493.09	HLA-B*08:01	This study
5	TDTPIVYNDRNL(LD)	N.512.12 (14)	NA	This study
6	EPIGSLAIEEAM	P.022.12	HLA-B*35:01	This study
7	YVYDHSGEAVK	P.111.11	HLA-A*03:01	This study
8	YVYDHSGEAVK	P.111.11	HLA-A*11:01	This study
9	GSAPISMGFR	P.169.10	HLA-A*03:01	This study
10	AEGGEIHEL	P.185.09	HLA-B*40:01	This study
11	FPKLGKTL	P.203.08	HLA-B*08:01	This study
12	KKQINRQN	P.350.08	NA	This study
13	DTGVDTRIW	V.282.09	NA	This study
14	AVRDLERAM	C.072.09	HLA-C*03:04	This study
15	AVRDLERAMTTLK	C.072.13	HLA-A*03:01	This study
16	KLWESPQEI	C.084.09	HLA-A*02:01	This study (4, 11, 21–24)
17	QEISRHQALGY	C.090.11	HLA-B*44:02	This study
18	GRLVPQVRVID	M.029.11	NA	This study
19	LLKEATEL	M.090.08	HLA-B*08:01	This study
20	GLNEKLVFY	M.106.09	HLA-B*15:01	This study
21	RLSDNGYYTV	M.164.10	HLA-A*02:01	This study (22)
22	FRSVNAVAF	M.180.09	HLA-C*07:02	This study
23	GKIIDNTEQL	M.203.10	NA	This study
24	KIIDNTEQL	M.204.09	HLA-A*02:01	This study
25	VIINDDQGLFKV	M.323.12	HLA-A*02:01	This study
26	DQGLFKVL	M.328.08	NA	This study
27	EPIRDALNAM	F.085.10	HLA-B*35:01	This study
28	SMLNSQAIDNLRA	F.140.13	HLA-A*02:01	This study
29	RQAGQEMILAV	F.165.11	HLA-A*02:01	This study
30	RQAGQEMILAV	F.165.11	HLA-B*15:01	This study
31	RITHVDTESY	F.268.10	HLA-B*15:01	This study
32	GPPISLERLDVGTN	F.449.14	NA	This study
33	RPGLKPDL	F.532.08	HLA-B*07:02	This study
34	LMIDRPYVL	H.030.09	HLA-A*02:01	This study (4, 11, 21, 23, 24)
35	AIYTAEIHK	H.064.09	HLA-A*03:01	This study
36	AIYTAEIHK	H.064.09	HLA-A*11:01	This study
37	LETRTTNQFL	H.172.10	HLA-B*40:01	This study
38	PTTIRGQFS	H.191.09	NA	This study
39	GMYGGTYLVEK	H.226.11	HLA-A*03:01	This study
40	KPNLSSKRSEL	H.236.11	HLA-B*07:02	This study
41	SMYRVFEV	H.250.08	HLA-A*02:01	This study (22)
42	APVFHMTNY	H.267.09	HLA-B*35:01	This study
43	APVFHMTNYLEQPVS(N)	H.267.15 (16)	NA	This study
44	IPYQGSGKGVSF	H.308.12	HLA-B*07:02	This study
45	IPYQGSGKGVSF	H.308.12	HLA-B*35:01	This study
46	IPPMKNLAL	H.456.09	HLA-B*08:01	This study
47	KVSPYLFTV	H.477.09	HLA-A*02:01	This study
48	AEVDGDVKL	H.502.09	HLA-B*40:01	This study
49	ARVPHAYSL	L.0032.09	HLA-C*07:02	This study
50	LLKKGNSLY	L.0109.09	HLA-B*15:01	This study
51	DIKEKVINL	L.0144.09	HLA-B*08:01	This study
52	ISKESQHVY	L.0213.09	HLA-B*15:01	This study
53	KESQHVYYL	L.0215.09	HLA-B*40:01	This study
54	KLIDGFFPA	L.0263.09	HLA-A*02:01	This study
55	YLKDKALA	L.0463.08	HLA-B*08:01	This study
56	KEIKETGRLF	L.0537.10	HLA-B*44:02	This study
57	AENLISNGIGKY	L.0560.12	HLA-B*44:02	This study
58	AQRLNEIY	L.0680.08	HLA-B*15:01	This study
59	YESGVRIASL	L.0760.10	HLA-B*40:01	This study
60	IVSSHFFVY	L.0824.09	HLA-B*15:01	This study
61	LPAPIGGMNY	L.0933.10	HLA-B*35:01	This study
62	SLMPEETLHQV	L.0971.11	HLA-A*02:01	This study
63	MPEETLHQVM	L.0973.10	HLA-B*35:01	This study
64	RPIYGLEV	L.1152.08	HLA-B*07:02	This study
65	SAVRIATVY	L.1237.09	HLA-B*35:01	This study
66	(K)KVDTNFIY(QQ)	L.1325.09 (L.1326.10)	NA	This study
67	HILAKSTAL	L.1434.09	HLA-B*08:01	This study
68	SMIDLVTKF	L.1443.09	HLA-B*15:01	This study
69	HYREVNLVY	L.1936.09	NA	This study
70	SQQGMFHAY	L.2076.09	HLA-B*15:01	This study

*^a^Code “x.(y)yyy.zz” of a measles virus peptide (pMV), in which x encodes the source protein (N, nucleocapsid; P, phosphoprotein; V, V protein; C, C protein; M, matrix protein; F, fusion protein; H, hemagglutinin; L, polymerase), (y)yyy encodes the position of the first aa in the protein, and zz represents the length of a peptide, respectively. Boxed epitopes represent identical or overlapping but differentially assigned epitope sequences*.

### MV Peptide Length Distribution and Dynamic Range

**Figure 1:**
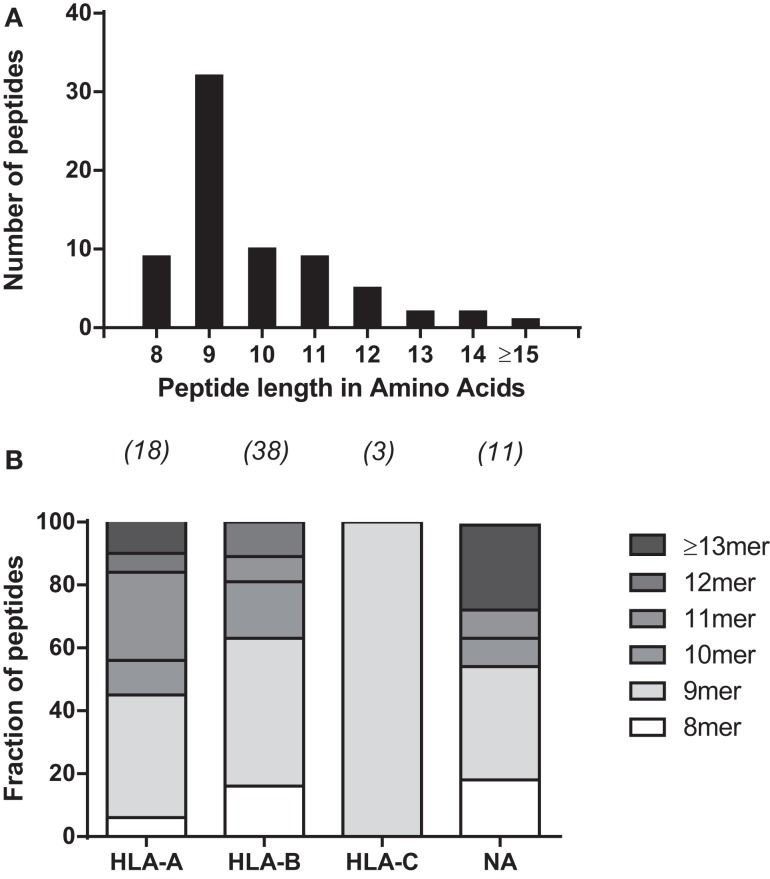
**General characteristics of HLA class I-eluted MV peptides**. **(A)** Distribution of peptide lengths for 70 unique HLApMV combinations eluted from the four BLCL. **(B)** Relative distribution of peptide lengths plotted per HLA locus for all unique HLApMV combinations eluted from the four BLCL Peptides ≥14 aa are grouped. Numbers represent total MV peptides per assigned group. NA, not assigned.

**Figure 2:**
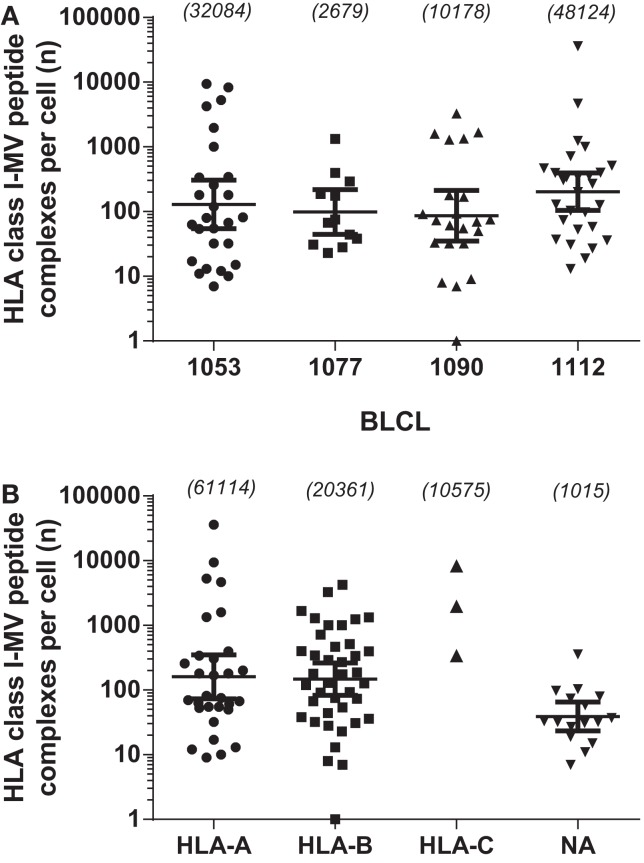
**Comparison of abundances of the 89 individually identified HLApMV combinations**. **(A)**: abundance of uniquely identified HLApMV combinations per BLCL. **(B)**: abundance of uniquely identified HLApMV combinations plotted per assigned HLA class I category. Each symbol represents an individual HLApMV combination from the summed 89 MV peptides identified in the four BLCL eluates (hence length variants shown individually). Numbers between brackets are total number of HLApMV complexes per BLCL **(A)** or total number of HLApMV complexes per locus **(B)**.

To further characterize the MV peptide species involved, we analyzed their general length and global expression rate. Uniquely presented MV epitopes ranged in length from 8 to 16 aa, with nonamers being most common (46%), followed by deca- (14%), octa-, and undecamers (both 2.9%) (Figure 1A). Based on peptide quantity, i.e., counting all copy numbers of HLApMV complexes of a given peptide length and summing these for all BLCL, nonamers are composed 81% of the total MV peptide repertoire studied, with percentages for octa-, deca-, undeca-, and dodecamers of 4, 1, 10, and 4%, respectively (data not shown). As depicted in Figure 1B, nonamers were dominant in the HLA-A and HLA-B sets of MV peptides, with other lengths occurring as well, including octamers and non-canonically long peptides (>11 aa). All three HLA-C-assigned peptides were nonamers. Furthermore, individual HLApMV combinations differed remarkably in their expression rate on BLCL, illustrating a dynamic range from as few as 1–10 to >35,000 cc (Figure 2A), while median expression rates were comparable between the four BLCL, i.e., 129, 99, 87, and 167 cc, respectively.

### HLA Class I Allele Usage and Hierarchy of Individual MV Peptides

**Figure 3:**
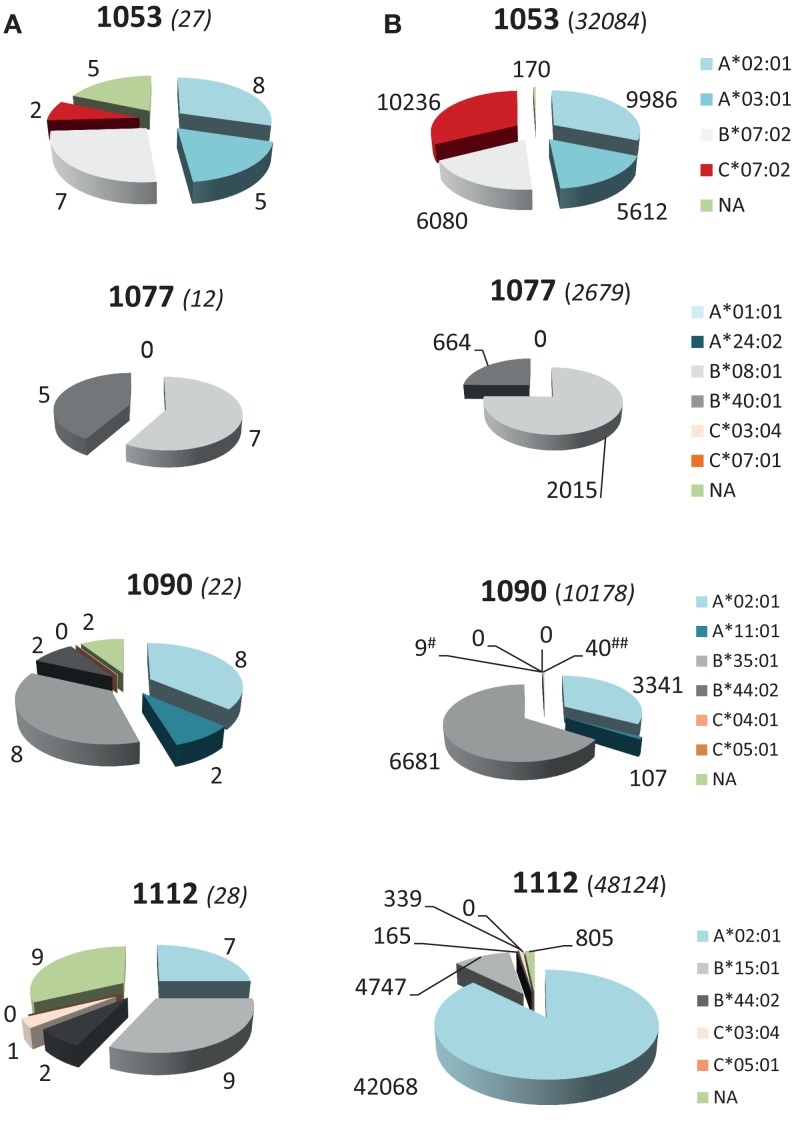
**HLA class I allele usage and hierarchy in number and abundance of total MV peptides**. **(A)** Number of unique HLApMV combinations assigned per allele per BLCL. **(B)** Distribution of total number of HLApMV complexes expressed per allele per BLCL. ^#^B*44:02; ^##^NA. Allele legends are given to the right. Numbers between brackets are total numbers of epitopes per BLCL **(A)** or total number of HLApMV complexes per BLCL **(B)**.

To study the role of the different HLA class I gene loci and alleles in MV peptide presentation in more detail, we analyzed the diversity and abundance of the MV peptides assigned per HLA class I molecule per individual BLCL. As is shown in Figure 3A, for each BLCL multiple MV peptides were identified yet these were not equally distributed over HLA class I molecules expressed. MV peptides were assigned to all six HLA-B alleles in the study, i.e., B*07:02 (*n* = 6 peptides), B*08:01 (*n* = 7), B*15:01 (*n* = 8), B*35:01 (*n* = 8), B*40:01 (*n* = 5), and B*44:02 (*n* = 3). For the other loci and their alleles, MV peptides were only allocated to HLA*A*02:01 (*n* = 11), A*03:01 (*n* = 5), A*11:01 (*n* = 2), C*03:04 (*n* = 1), and C*07:02 (*n* = 2). The 11 NA MV peptide species, three of which were confirmed on two different BLCL, and one on three different BLCL, respectively, were relatively low-abundant (geomean of 39 cc, Figure 2B). HLA-A and HLA-B-assigned MV peptides generally had a higher expression rate (geomean of 160 and 148 cc, respectively). Of interest, HLA-A-associated MV peptides reached highest absolute numbers than HLA-B-associated MV peptides, with 36,108 cc observed for the most abundant and dominating peptide in HLA-A*02:01 (C.084.09 in BLCL1112) versus 4,219 cc for the most abundant peptide in HLA-B*07:02 (N.411.10 in BLCL1053), respectively. High abundance was also seen in HLA-C: one (of three) identified peptides, M.180.09, was found at 8,266 cc in HLA-C*07:02 (BLCL1053). Notably, BLCL1077 had a relatively low yield of MV peptides, both in number (Figure 3A) and abundance (Figure 3B), and only yielded HLA-B-associated MV peptides (though not being homozygous for HLA-B).

### MV Proteome Coverage and Strain Variability of HLA Class I-Selected Epitope Regions

**Figure 4:**
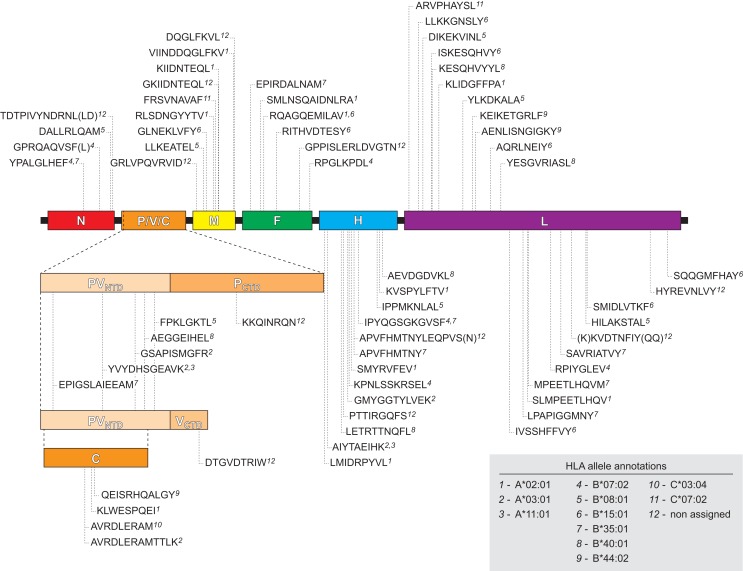
**Schematic location of the naturally processed and presented HLApMV epitopes in the MV proteome**. Indicated are epitope sequences, assigned HLA restricting elements and positions of the first amino acids of the MV epitopes, relative to the MV gene segments encoding N (nucleocapsid), P/V/C [three unique proteins encoded differently from the *P* gene as indicated: phosphoprotein (P) and V protein, sharing an N terminal domain (PV_NTD_) and each having a unique C terminal domain (P_CTD_ and V_CTD_) via differential RNA editing, and C protein expressed from an alternative open reading frame, respectively (25)], M (matrix protein), F (fusion protein), H (hemagglutinin), and L (polymerase), respectively.

**Figure 5:**
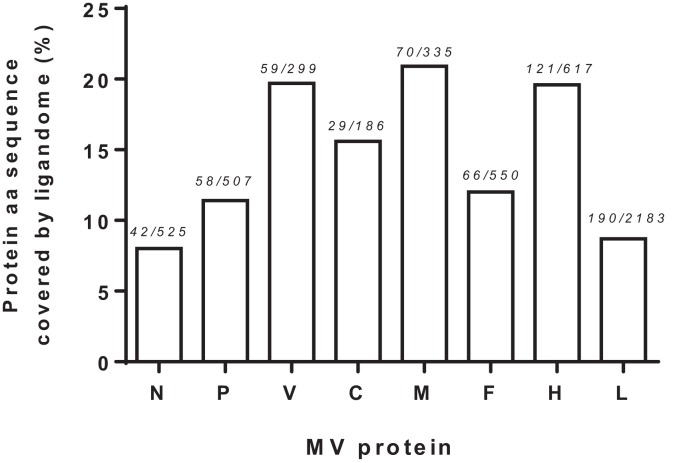
**Measles virus protein sequences covered by the identified HLA class I ligandome**. Visualization of relative MV protein sequence coverage by the cumulative aa of the HLApMV sequences identified, analyzed per protein. Numbers indicate summed length of the HLApMV ligandome in aa divided by the protein length in aa.

We next examined the viral proteome represented by the 70 identified HLApMV combinations. In 11 cases, (near) identical or overlapping MV peptide sequences were assigned differently in different cell lines, e.g., to two separate HLA class I molecules (*n* = 8) or to one HLA class I allele and the other remaining NA (*n* = 3), Table S2 in Supplementary Material). Taking these into account, the HLApMV panel involved 59 different source regions, which were scattered throughout the MV proteome, implying all eight MV proteins (Figure 4). MV-P and MV-V are expressed from the same *P* gene segment by a process called RNA editing (25) and share their N terminal part (aa 1–231). As indicated, this shared N terminal domain yielded five MV peptides, hence both proteolysis of MV-P and of MV-V could account for these. The unique C-termini of MV-P and MV-V also generated one additional HLApMV species each (Figure 4). Of the total expressed MV proteome (5,202 aa), the identified HLA class I-presented MV ligandome comprised 12.2% (*n* = 635 aa). The relative sequence coverage was highest for MV-M (7 epitopes, representing 70 aa of 335 aa, 20.9%) and lowest for MV-N (4 epitopes, representing 42 aa of 525 aa, 8.0%) (Figure 5).

Since molecular evolution of MV genomes has been considered slow but nevertheless subject to selective pressure of immune responses (2), we performed a preliminary analysis of the occurrence of non-synonymous substitution rates (*dN*) in codons from all MV epitope regions relative to the occurrence of synonymous substitution rates (*dS*) and the overall genomic evolution in their source proteins using a database of 58 full-length publicly available unique MV genomes (listed in Table S1 in Supplementary Material) and SNAP v2.1.1 software (see Materials and Methods). Eleven of the 59 epitope regions did not contain any codons with non-synonymous substitutions, the other 48 regions containing at least one codon with a non-synonymous substitution (median 2, range 1–7) (Raw data showing the variability analysis of the full-length proteins are given in Table S3 in Supplementary Material, a summary of the microvariation in MV epitopes is given in Table S4 in Supplementary Material). Epitopes lacking non-synonymous substitutions (*dN* = 0) were mostly from MV-L (9/11), with one other epitope from MV-N and one from MV-F (Table S4 in Supplementary Material). In 88 of 132 codons representing epitope regions displaying aa variation, *dN* was greater than *dS* (*dN* > *dS*). When omitting epitope regions of the genomically conserved MV-L from this list, we noted a trend that HLA-A associated epitope regions had a higher proportion of codons with *dN* > *dS* of their total number of codons than HLA-B-associated epitope regions (22.2 versus 14.1%, *p* = 0.0795). Moreover, for epitopes with abundances of ≥1,000 cc, the proportion of *dN* > *dS*-codons was significantly higher than epitope regions with abundances <1,000 cc (28.9 versus 15.2%, *p* = 0.0061) (Table S4 in Supplementary Material).

Codons with *dN* > *dS* were observed throughout the MV proteome, in some protein sequences more frequently than in others and not limited to the epitope regions identified in this study (Table S3 in Supplementary Material). Our list of epitope regions likely underrepresents the full MV immunopeptidome, due to sensitivity limits of MHC peptide elution methods and the selective panel of HLA class I alleles investigated. A preliminary comparison of the proportion of codons with *dN* > *dS* in regions representing epitopes elucidated in this study (including MV-L epitopes) versus regions not representing epitopes indicated an increased occurrence of codons with higher *dN* than *dS* in the current epitope list (data not shown).

## Discussion

Cutting edge mass spectrometric analysis has allowed us to extend our knowledge on the selection and presentation of MV-derived peptides in the context of a broad set of HLA class I alleles. Seventy unique HLA class I peptide combinations representing 59 viral epitope regions were identified as candidate targets for the CD8^+^ T cell response, mostly unknown to date (4, 11, 21–24). This is an unprecedented large set of naturally presented HLAp revealed for a single virus. This comprehensive MV epitope landscape allowed us to make two major observations. The first, in line with our hypothesis, is that the most abundant peptide species in our study were presented by HLA-A alleles, while HLA-B alleles displayed an overall more diverse set of MV peptides. The HLA-A*02:01-assigned MV-C.084.09 peptide was found in all three HLA-A*02:01 expressing MV-infected BLCL and appeared at high density, up to 36,000 cc, in accordance with earlier observations (11). The second most abundant HLA-A-associated epitope was MV-P.111.11, expressed by HLA-A*03:01 (5,279 cc). Remarkably however, and not corroborating with an HLA-A exclusive claim on supra-abundance, the HLA-C*07:02-assigned epitope M180.09 was also found at a high copy rate (>8,000 cc), for the first time implying the HLA-C locus in the presentation of epitopes at such remarkably high level of expression. This seems even more interesting in view of the substantially lower expression rate measured for HLA-C molecules compared to HLA-A and -B molecules on BLCL or PBMC, being up to 12- to 13-fold lower depending on steady state or infection conditions (26). Although we have not assessed locus-specific expression of HLA class I molecules in a quantitative manner in our study, we could not observe overall changes in HLA class I expression upon MV infection of BLCL (see Materials and Methods).

No other HLA-A nor B or C alleles in our study were implied in supra-abundant expression rates >5,000 cc, yet HLA-B*08:01, HLA-B*15:01, and HLA-B*35:01 were found among the HLA class I alleles expressing several MV epitopes in the abundance range of 1,000–5,000 cc (Table S2 in Supplementary Material). Although still in a selection of HLA class I molecules, these observations might indicate that alleles from all three HLA-A, -B, and -C loci can, in principle, contribute to abundant viral epitope expression (≤5,000 cc) at the cell surface, but with certain HLA-A, and -C alleles potentially driving supra-abundance for particular epitopes. Although we have not studied the new set of epitopes for T cell immunogenicity yet, based on earlier work it can be envisaged that supra-abundant epitopes may lead to dominant early expansion of CD8^+^ T cells which however decline below detection limit in the memory phase, as was found for the HLA-A*02:01 binding MV-C.084.09 peptide (4). Other HLA class I alleles that presented peptides at a supra-abundant rate (>5,000 cc) were HLA-A*03:01 and HLA-C*07:02, which like HLA-A*02:01 are globally highly prevalent alleles^3^, implying that supra-abundance of MV epitopes is possible across populations. This feature could contribute to measles-associated immunosuppression by strong expansions in the specific CD8^+^ T cell compartment in large patient groups (5), wiping out not only large fractions of primary infected lymphocytes but also any target cell licensed to be killed by presenting peptide shed from infected cells. On the other hand, the more moderately expressed epitopes in the MV peptides landscape may underlie memory CD8^+^ T cell responses that do mediate effective long-lived CD8^+^ T cell memory. HLA-B molecules, being the most polymorphic of the HLA isotypes, may in particular contribute to this repertoire by accommodating a more diverse set of epitopes, as was shown for *self*-derived peptides (13) as well as for MV peptides (this study). Whether this leads to more immunodominant MV-specific HLA-B-restricted CD8^+^ T cell responses in the memory phase, as has been seen in various other infectious models (27–30), remains to be elucidated in T cell studies using blood samples taken longitudinally after MV infection or immunization.

Hence, the extreme high and low abundance ends found in the MV epitope landscape observed could well contribute to the measles paradox of respective immunosuppressive and immunity mechanisms, generated in parallel. Notably, high copy number presentation by HLA-A*02:01 was recently suggested to play a role in the low-avidity non-protective cytotoxic T cell responses against the M1_58–66_ Influenza peptide GILGFVFTL (31). Such stealth mechanism may even be more pronounced for the C.084.09 KLWESPQEI epitope of MV, poising the HLA-A*02:01 landscape (24) and being far more abundant than GILGFVFTL. It would be of interest to investigate whether the non-responsiveness of CD8^+^ T cell responses observed against KLWESPQEI during convalescence of measles (4) is related to low TCR avidity or to activation induced cell death, which could occur after inappropriate or prolonged TCR stimulation by high-dose peptide (32). Another point to be elucidated is how protective immune responses to less abundant peptides can be generated in the presence of overwhelming amounts of stealth peptides, as T cells compete for interaction with antigen-presenting cells (33), but temporal regulation of epitope expression could play a role (24).

The MV peptide set contained, apart from mostly peptides with a normal length distribution (8–12 aa), a few low-abundant, non-canonical 14, 15, and 16-mers. These all failed to be assigned to a particular HLA class I allele. In general, peptides longer than 13 aa in length seem to follow binding motifs less well (Meiring HD, unpublished observations). In line with this, the long MV epitope sequence H.267.15, categorized as NA in BLCL1112, was also detected as a shorter length variant in another BLCL that was assigned to an HLA allele (H.267.09, assigned to HLA-B*35:01 in BLCL1090). Also F.452.14, NA in BLCL1112, was assigned as a shorter peptide in the literature [F.452.10, presented by HLA*02:01 (22)].

The second major observation was that a substantial part (12.3%) of the MV proteome was represented in the overall elucidated HLA class I-presented viral peptidome, with MV-P/V, C, M, and H protein sequences being relatively highly sampled by HLA class I. Moreover, the majority of epitope regions, except in MV-L, had genomic variation with aa substitutions occurring at a higher rate than the silent nucleotide substitutions (*dN* > *dS* sites), potentially representing selective pressure. Of the 18 codons with the highest non-synonymous mutation rates (*dN* ≥ 0.5) in the full length viral genome (*MV-L* codons excluded), 11 were within epitope regions, 8 of which were located in *MV-P/V/C, -M, and -H* genes (Table S4 in Supplementary Material). Earlier, based on 162 *H gene* sequences, Saitoh et al. found evidence for significant positive selection pressure on aa H.476 (F- > L), adjacent to the HLA-A*02:01-eluted H477.09 (KVSPYLFTVA), to be positively selected via an unknown mechanism. In our preliminary selection of 58 MV genomes, the H.476 codon had a lower *dN* rate (0.11, *dS* = 0) than H.481 (Y- > N), a codon within the H477.09 epitope (*dN* = 0.57; *dS* = 0.03), implying that molecular evolution of this epitope may also be significant when tested in more strains, and could well involve T cell mechanisms. For another H-derived epitope with high codon variability, the HLA-A*02:01-presented octamer H.250.08 (SMYRVFEV, Y- > H, *dN* = 0.5), CD8^+^ T cell reactivity has been implied in HLA-A2 positive vaccinees and patients using a synthetic decamer peptide (SMYRVFEVGV) comprising the naturally occurring sequence (22). We observed that epitope abundance (≥1,000 cc) rather than the HLA class I locus of HLApMV was involved in driving selection of coding mutation in codons. To further evaluate the significance of epitope abundance or the role of HLA-A versus-B presentation on codon evolution, more insight into the full breadth of human HLA class I-presented MV epitope regions is required, as well as a larger set of non-redundant MV genome sequences, ideally from strains obtained directly from acute cases, for codon analysis (2, 3).

With the use of front line mass spectrometry to detect viral HLA class I epitopes we have begun to unravel the landscape of naturally expressed MV epitopes. Based on our study, it can be concluded that MV-specific CD8^+^ T cells may encounter a widely divergent epitope repertoire at the cell surface of an infected target cell, including stealth peptides such as the HLA-A*02:01 presented C.084.09 KLWESPQEI, and also multiple more modestly expressed peptides. Subsequent molecular interactions may then be key to either programing for a long-term maintained response or the induction of a vigorous short-term response that may be immunosuppressive for the host and immunoselective for viral variants.

## Conflict of Interest Statement

The authors declare that the research was conducted in the absence of any commercial or financial relationships that could be construed as a potential conflict of interest.

## Footnotes

^1^www.uniprot.org

^2^http://www.hiv.lanl.gov/

^3^www.allelefrequencies.net
