# Supplementary material for: Measles Virus Epitope Presentation by HLA: Novel Insights into Epitope Selection, Dominance, and Microvariation
Source: Front Immunol. 2015 Nov 2;6:546. doi: 10.3389/fimmu.2015.00546 (PMC4629467; doi:10.3389/fimmu.2015.00546)
Supplement: Supplementary file 5 [file Image_1.PDF]

Supplementary Figure S1. **Nanoscale LC-MS identification of a MV peptide from a MV-infected BLCL**

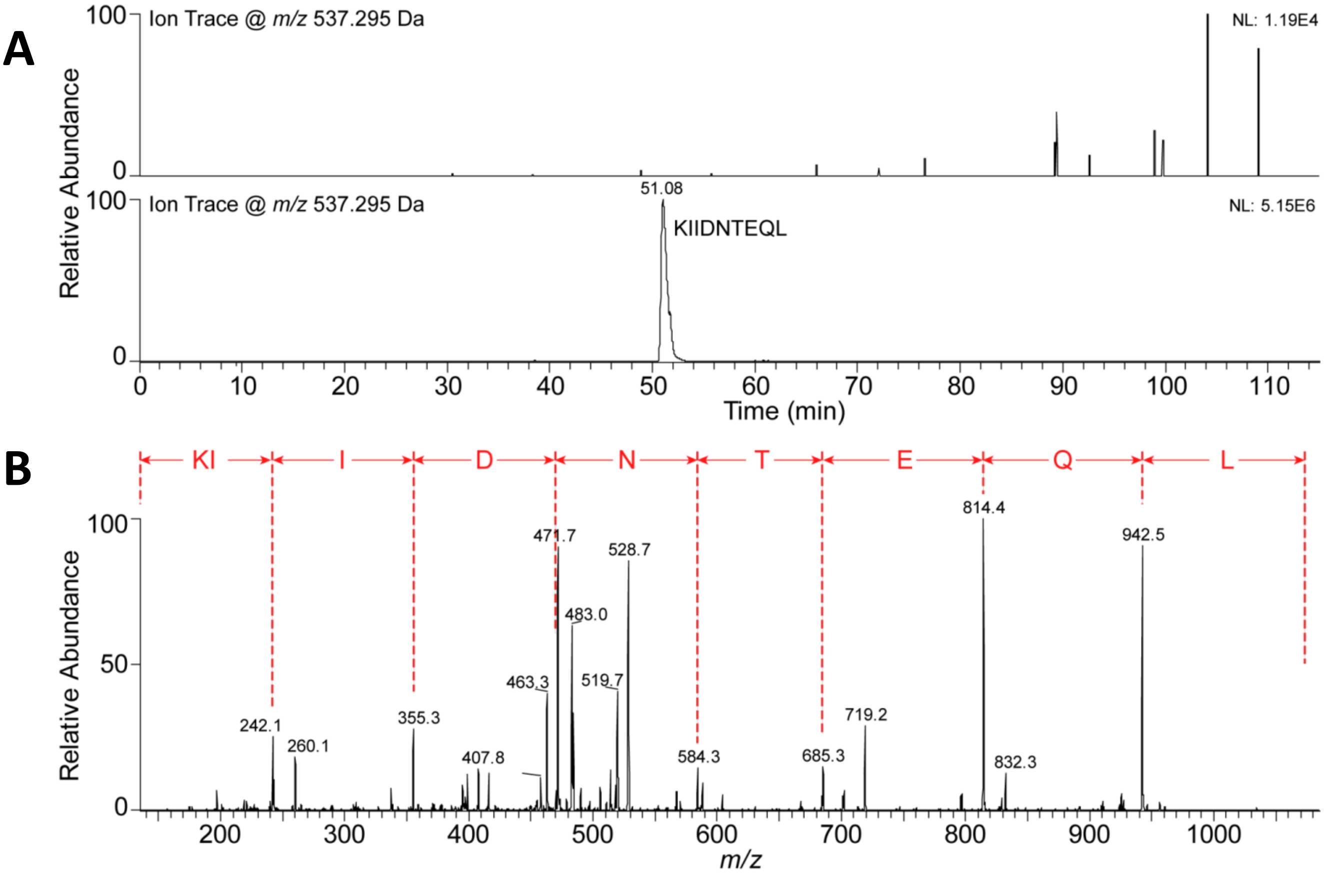

Legend. SCX fractions of the HLA class I eluate from MV-infected and un-infected BLCL1112 were subjected to nanoscale LC-MS analysis. **A**. Ion traces of a selected ion of a MV peptide at MH2+ at m/z 537.295 in the uninfected control sample (upper trace) and in the MV-infected sample (lower trace). **B**. Deconvoluted MS/MS spectrum of this peptide identifies its sequence (b-type ions series) as KIIDNTEQL, matching aa residues 204-212 from MV-M (M.204.09), and the summed signal of this peptide corresponds to a semi-quantitated density of 4,666 cc. M.204.09 was also identified in the sample from MV-infected BLCL1053 (not illustrated).
